# Strengthening healthcare through family medicine training in The Gambia: The journey so far

**DOI:** 10.4102/phcfm.v16i1.4446

**Published:** 2024-04-25

**Authors:** Abraham N. Gyuse, Iorfa Tor-Anyiin, Joshua P. Mwankwon, Ousman Nyan, Horeja Saine

**Affiliations:** 1 Department of Family Medicine, Faculty of Clinical Sciences, University of Calabar, Calabar, Nigeria; 2 Department of Family Medicine, Edward Francis Small Teaching Hospital, Banjul, The Gambia; 3 Department of Family Medicine, Federal Medical Centre, Makurdi, Nigeria; 4 Department of Family Medicine, University of Calabar Teaching Hospital, Calabar, Nigeria; 5 Department of Internal Medicine, Edward Francis Small Teaching Hospital, Banjul, The Gambia; 6 Department of Nursing, Edward Francis Small Teaching Hospital, Banjul, The Gambia

**Keywords:** essential health, strengthen, family medicine, journey, Gambia

## Abstract

According to the World Health Organizations (WHO) family medicine forms the bedrock upon for accessible, affordable and equitable healthcare for any country. The need for family doctors is more acute for low income countries like The Gambia. More so that The Gambian health infrastructure is suboptimal and appropriate health personnel is low. This is worsened by brain drain leading to poor health indices. Despite these challenges and more, the department of Family Medicine was accredited for training in the Gambia with improved infrastructure (at the training centre), with 7 residents. Though there are still challenges there are also opportunities and strengths. There is therefore hope that the right personnel will be produced for an improved Gambian health system.

## Introduction and overview of The Gambia

The Republic of The Gambia is the smallest country within mainland Africa. It is nearly completely surrounded by Senegal, except for its western coast on the Atlantic Ocean. It is home to several ethnic groups, including Mandinka, Fula and Wolof, and has a population of approximately 2.4 million people. Its economy is reliant on agricultural exports and tourism.^1,2^

The Edward Francis Small Teaching Hospital (EFSTH) located in Banjul (Capital of The Gambia) established in 1894 as Royal Victoria Hospital, transformed into a teaching hospital for the School of Medicine and Allied Health Sciences of the University of The Gambia in 2002. It is the largest referral centre and the only tertiary and teaching hospital in the country.^3^

The 2021 country statistics shows that the healthcare infrastructure consists of the following: one teaching hospital, one specialist hospital, four district hospitals, six major health centres and 40 minor health centres. The major health centres are equipped to provide comprehensive emergency care, while minor health centres are equipped to provide basic emergency obstetrics and neonatal care. The public sector accounts for 88.97% of the total bed capacity in the country, while private-owned facilities and non-governmental Organisation (NGO) (owned) hospitals account for 11.03% of the bed capacity.^4^

## Background and context

The Gambia has always endured a paucity of doctors. The medical school and several technical assistance programmes that provide doctors have added too few, mainly transient doctors, to make any meaningful impact on primary health care (PHC) delivery. Consequently, nurses lead all district hospitals and health centres. Occasionally junior doctors are posted there without appropriate supervision. The doctors commonly leave The Gambia in search of greener pastures and/or specialist training, which is not available in The Gambia.^5^

This paucity of doctors could be responsible for the poor health indices of The Gambia as shown by the 2019–2020 Demographic Health Survey: neonatal mortality 29/1000 live births; infant mortality 42/1000 live births; under-5 mortality rate 56/1000 live births; maternal mortality ratio 289/100 000 live births.^6^ It is noteworthy, however, that these values are an improvement on the indices prior to the 1980s implementation of PHC policies.^7^

It is obvious that the need for training and retention of doctors within The Gambia cannot be overstated. This need is most acute for family medicine, which provides doctors with the broad array of competences fit for manning regional hospitals. The effort to establish this training was given a boost by the visit of the West African College of Physicians (WACP) to the EFSTH in 2017.

The Establishment of family medicine in the Gambia was not without challenges. More so that it was previously unknown at the institutional level and was not taught at the undergraduate level. There is only one known Gambian who is a Fellow of WACP in family medicine. Hence to establish family medicine training in The Gambia, the EFSTH recruited Prof. Abraham N. Gyuse and two other consultants from Nigeria with the aid of the World Bank. The Department of Family Medicine was established on 21 September 2021 with seven residents amidst severe infrastructural and workforce deficits. Despite these constraints, team guided by Prof. Gyuse led the hospital to attain infrastructural and functional standards worthy of accreditation for training by the WACP in April 2023.

These included a massive infrastructural upgrade of the former polyclinic at EFSTH to serve as the main training complex. The clinical services at the complex are now physician-led and structured to meet global best practices and accreditation standards. The clinical environment has well-furnished consultation rooms, a resuscitation bay, procedure room and an observation ward. The complex also has a seminar room and a resource room (equipped with computers and 24-h internet services). The department also collaborates with the Clinical Service Department of Medical Research Council Unit (MRC) Gambia for further clinical exposure and research.

The advocacy and service delivery efforts of the new department have started yielding some fruit, with increasing awareness of family medicine at the institutional level and in the general Gambian public. Other specialists are also recognising the role of the specialty in healthcare delivery. The allure of the specialty has attracted four junior doctors who recently passed the family medicine primary examination of the WACP, thus can start specialist training.

It is hoped that once the Family Medicine Training Programme begins to graduate family physicians, they will be posted to all district hospitals in sufficient numbers with the needed skills-set to manage this level of the health services. This in turn should translate to improved patient outcomes country-wide, improved referral quality, improved patient satisfaction and consequently health indices. This should further convince policymakers, the medical fraternity and the health-seeking public on the positive role of family medicine in improving and strengthening the Gambian health services.

Challenges, however, still exist and include sustainability of good remuneration for trainers and residents; unrealistic expectations by the health-seeking public, health authorities and other specialists. The rural component of the training has not been provided with the necessary infrastructure for its take-off, and the University of the Gambia has not incorporated family medicine into her undergraduate training. This is summarised in a strengths, opportunities, weaknesses and threats (SWOT analysis) shown in Table 1.^8^

**TABLE 1:** Strengths, opportunities, weaknesses and threats (SWOT analysis) of the current scenario of family medicine, primary care and primary health care in The Gambia according to the World Health Organization’s Four (4) Core strategic levers.

WHO core strategic lever	Strengths and opportunities	Weaknesses and threats
Political commitment and leadership	MoH and government promoting PG training in FM.	Poor general understanding of family medicine by policy makers, other medical disciplines in the country and the general population
	Hospital management also enthusiastic, along with other institutional leaders (HODs and other trainers).	Push-back from other disciplines and paramedical staff
		No clear sustainability strategy for FM training and practice in place
Governance and policy frameworks	Enthusiasm of government through MoH and hospital board	Poor understanding of primary health care-oriented services by health managers with focus on specialist care
	Recruit from subregion and obtain accreditation from the regional training college	Inability of policy makers to differentiate family medicine and/or primary care from other clinical specialties and areas like community health.
	Proposed formation of the Gambian College of Physicians and Surgeons with FM as one of the foundation faculties	No clear policy on integrating family medicine and/or primary care into the health system
Funding and allocation of resources	Funding through the World Bank and other donor agencies	A lack of financial incentives to start PG programme; no career path after specialisation.
	Many donor agencies already providing financial assistance in the country	Poor remuneration affecting availability of trainers or teachers
		Disproportionate allocation of resources to non-essential areas to the detriment of clinical and training needs
Engagement of communities and other stakeholders	Expressed interest in family medicine by Gambian Regional Health teams and district hospitals.	There is no clear community understanding of health sector work structure and operation
	Long-standing ties with MRC Gambia unit in clinical services delivery and policy formulation.	
		Poor integrated work culture among essential health workers.
		Common and similar cultural and religious practices of the health-seeking public.
		No integrated leadership of the health team at all levels

*Source*: Extracted and modified from: WHO. Operational framework for primary health care: Transforming vision into action [homepage on the Internet]. 2020 [cited 2023 Jul 06]. Available from: https://apps.who.int/iris/bitstream/handle/10665/337641/9789240017832-eng.pdf?sequence=1&isAllowed=

WHO, World Health Organization; MoH, ministry of health; PG, postgraduate; FM, family medicine; HOD, head of department; MRC, Medical Research Council Unit.

## Conclusion

The Gambian government is on the right trajectory to improving the country’s primary care services through Family Medicine training. It is hoped that this can be sustained and challenges addressed such that in the near future qualified specialist family doctors will be able to manage the district health services and continue with the training of others, leading to an improved primary health care delivery in The Gambia.
